# Surgical outcomes for colon and rectal cancer over a decade: results from a consecutive monocentric experience in 902 unselected patients

**DOI:** 10.1186/1477-7819-5-73

**Published:** 2007-07-04

**Authors:** Bruno Andreoni, Antonio Chiappa, Emilio Bertani, Massimo Bellomi, Roberto Orecchia, MariaGiulia Zampino, Nicola Fazio, Marco Venturino, Franco Orsi, Angelica Sonzogni, Ugo Pace, Lorenzo Monfardini

**Affiliations:** 1 Dept. of General Surgery, European Institute of Oncology, University of Milano, Italy; 2 Division of Radiology, European Institute of Oncology, University of Milano, Italy; 3 Division of Radiotherapy, European Institute of Oncology, University of Milano, Italy; 4 Division of Oncology, European Institute of Oncology, Milano, Italy; 5 Division of Anaesthesiology, European Institute of Oncology, Milano, Italy; 6 Division of Pathology, European Institute of Oncology, Milano, Italy

## Abstract

**Background:**

This study evaluates the surgical morbidity and long-term outcome of colorectal cancer surgery in an unselected group of patients treated over the period 1994–2003.

**Methods:**

A consecutive series of 902 primary colorectal cancer patients (489 M, 413 F; mean age: 63 years ± 11 years, range: 24–88 years) was evaluated and prospectively followed in a university hospital (mean follow-up 36 ± 24 months; range: 3–108 months). Perioperative mortality, morbidity, overall survival, curative resection rates, recurrence rates were analysed.

**Results:**

Of the total, 476 colorectal cancers were localized to the colon (CC, 53%), 406 to the rectum (RC, 45%), 12 (1%) were multicentric, and 8 were identified as part of HNPCC (1%). Combining all tumours, there were 186 cancers (20.6%) defined as UICC stage I, 235 (26.1%) stage II, 270 (29.9%) stage III and 187 (20.6%) stage IV cases. Twenty-four (2.7%) cases were of undetermined stage. Postoperative complications occurred in 38% of the total group (37.8% of CC cases, 37.2% of the RC group, 66.7% of the synchronous cancer patients and 50% of those with HNPCC, p = 0.19) Mortality rate was 0.8%, (1.3% for colon cancer, 0% for rectal cancer; p = 0.023). Multivisceral resection was performed in 14.3% of cases. Disease-free survival in cases resected for cure was 73% at 5-years and 72% at 8 years. The 5- and 8-year overall survival rates were 71% and 61% respectively (total cases). At 5-year analysis, overall survival rates are 97% for stage I disease, 87% for stage II, 73% for stage III and 22% for stage IV respectively (p < 0.0001). The 5-year overall survival rates showed a marked difference in R0, R1+R2 and non resected patients (82%, 35% and 0% respectively, p < 0.0001). On multivariate analysis, resection for cure and stage at presentation but not tumour site (colon vs. rectum) were independent variables for overall survival (p < 0.0001).

**Conclusion:**

A prospective, uniform follow-up policy used in a single institution over the last decade provides evidence of quality assurance in colorectal cancer surgery with high rates of resection for cure where only stage at presentation functions as an independent variable for cancer-related outcome.

## Background

Although colorectal cancer (CRC) must be seen as a tumour biological entity, the prognosis for colon cancer and rectal cancer individually differs considerably. The most important reason is certainly the great difference in loco-regional tumour failure, which is significantly higher for rectal cancer. In addition, adjuvant therapy regimens for colon cancer and rectal cancer as well as neoadjuvant radio/chemotherapy in selected patients with rectal cancer differ substantially. Based on the International Union Against Cancer (UICC)/American Joint Committee on Cancer (AJCC) tumour staging system [1-5], complete tumour removal (R0 resection) [1-7], is essential for local tumour control and long term survival.

The aim of this study was to review a large consecutive series of colorectal cancer patients prospectively followed between 1994 and2003. Morbidity and long-term survival after colorectal cancer surgery in relation to stage and radicality as well as after multivisceral resection, were analysed.

## Methods

### Patients

Between January 1994 and December 2003, a total of 902 patients were treated for primary colonic or rectal cancer. Patients' median age was 63 ± 11 years (range 24–88 years). There were 489 men and 413 women. Tumours were classified as rectal when adenocarcinomas were located within 12 cm above the anal verge with rigid proctoscopy. Multicentric colorectal cancers were staged according to the most advanced of the tumours.

Data concerning clinico-pathological staging and postoperative course were collected prospectively using a hospital tracking system based on ICD-coding for colorectal cancer with all Histologically confirmed cases were included. Eight hundred and seventy-three patients (96.8%) underwent elective surgery after mechanical bowel preparation using phosphates in 4 L of water, 29 patients (3.2%) were submitted to emergency surgery without any bowel preparation. Among these there were 14 patients (1.6%) presenting with secondary large bowel perforation, 12 (1.3%) with obstructing tumour and 3 (0.3%) with actively bleeding rectal cancer.

### Preoperative staging

Preoperative staging was performed by abdominal ultrasound, thoraco-abdominal CT scan, abdominal magnetic resonance imaging and endoscopic ultrasound as single modalities or in combination depending on their availability and the surgeon's preference. All patients had at least one form of preoperative imaging for staging purposes.

### Surgical procedure

Tumour resections were performed *en bloc *after ligation of the segmental vessels, followed by lymph node dissection. Anastomoses were established by stapling devices, usually performed in an end-to-end fashion for left, transverse and rectal resections and were termino-lateral for right colon resections. In selected cases we performed anastomoses according to Knight-Griffen's technique [8]. Staplers were routinely used. Coloanal anastomoses were performed usually combined with a protective loop ileostomy.

Standard resections were defined as tumour resections including standard lymph dissections restricted to the tumour-bearing bowel section. Multivisceral resections were defined as "organs or structures adherent to the tumour with a need for *en bloc *removal to obtain a curative situation". These multivisceral resections were classified according to the organ site (genitourinary system, liver, small bowel) or as "various" (i.e.: abdominal wall, large bowel). All operations were performed by the same surgical team (BA, RB, AC, FL, SP, UP, EB); each of whom had undergone postgraduate specialist training in colorectal surgery.

Peroperative morbidity and mortality were registered following 30 days after surgery.

### Pathology

Tumours were staged histopathologically and clinically according to the TNM/Dukes/UICC system [9].

The radicality of the surgical procedure performed was classified as "curative" (R0, no tumour left behind microscopically at resection margins); "questionably curative" (R1, tumour left behind microscopically at resection margins, or any other "Gray zone" situation that would question a curative operation, such as suspect but unproven metastases); "palliative" (R2, macroscopical tumour left behind); or "unresectable" [4].

### Adjuvant/neo-adjuvant treatments

Rectal cancer patients with locally advanced tumours (T3, T4, or N positive) defined by preoperative staging investigations, received bifractionated accelerated radiotherapy for a maximum of 41.6 Gy or conventional radiotherapy for a total of 50.4 Gy and concomitant chemotherapy (within clinical studies with regimens containing 5-fluorouracil, folinic acid, oxaliplatin, methotrexate or raltitrexed). Patients who did not receive neoadjuvant therapies with a pathological staging pT3-4 N0 and pT1-N1-2 cases received postoperative radio-chemotherapy. For patients undergoing Mayo Clinic [10] or Machover regimens [11] for 6 cycles "sandwich" schedules were used (2 cycles CT→RT→4 cycles CT). Patients with a central venous system underwent continuous infusion chemotherapy with 5-FU concomitant with radiotherapy. The treatment will last for 4–6 months. Chemotherapy regimens as described were employed in an adjuvant setting for patients who underwent preoperative bifractionated radiotherapy or for C and D colon cancer patients. UICC stage T3N0 colon cancer patients were candidate for the same chemotherapy regimens according to the presence of risk factors such as dedifferentiated tumour or vascular invasion after counselling with medical oncologist.

The indication for additional treatment was assessed either adjuvantly in curatively resected lesions in lymph node-positive stages, or palliatively-resected cases for stage IV or unresectable lesions. Liver or lung metastases were resected if, an R0 situation was expected after resection. If in fact an R0 situation was obtained, these patients were classified as R0 patients; otherwise they were R1 or R2. Metastases were resected in 32 of 188 UICC stage IV patients (17%) with synchronous liver metastses and without additional metastatic spread. Of all 902 patients, 560 (62.1%) underwent additional treatment: chemotherapy in 438 (48.6%), radiotherapy in 4 (1%) and radio-chemotherapy in 118 (13.1%) mainly for stage II or III rectal cancer (111/122 patients, 91%, who underwent postoperative radiotherapy). Eighty-four patients affected by rectal cancer underwent neoadjuvant radiotherapy, in 36 of them (75%) associated with chemotherapy.

### Follow-up

Patients were followed after curative resction using a follow-up program. This program consisted of three appointments per year (years 1–3), two appointments per year (years 4–5), and yearly appointments (years 6–10). All appointments included clinical evaluation, carcinoembryonic antigen (CEA) serum test, faecal blood test, abdominal ultrasonography (months 8, 20, 30, 42, and 54), chest radiography (months 12, 24, 36, 48, and 60), colonoscopy or double-contrast enema (months 12, 24, 48), rectal endoscopy (months 12, 24, 48), computed tomography (CT) (months 4, 16, 30, 42, and 54), and MRI where appropriate. When loco-regional recurrence was suspected, positron emission tomography (PET) scanning was used if available. Loco-regional recurrence was defined as histologically- or radiologically-proven disease presenting within the field of previous surgery. Thirty-two patients (3.5%) were lost to follow-up. Mean length of follow-up for 870 patients was 42 month (range 6–108).

### Statistics

Analysis was performed using a statistical software package (SPSS, Chicago, IL advanced model statistical package). Comparison of variables was performed with the Chi-square test and the Student's t-test for categorical and continuous variables, respectively. Actuarial local recurrence and survival were analysed using the Kaplan-Meier method with comparisons between groups being made with the log-rank test. Multivariate analysis was performed using the Cox proportional Hazard model where p values < 0.05 were considered to be significant.

## Results

### Operative findings

A total of 902 patients with colorectal cancer were included in the analysis. Among the lesions, 476 (53%) were localized in the colon, 406 (45%) in the rectum, 12 (1%) were multicentric and 8 (1%) were identify as a part of HNPCC. Surgical procedures are listed in Table 1. Among these, there were 12 patients whose tumour was excised transanally, 7 patients submitted to low anterior resection or Miles operation who showed a complete response to neoadjuvant RT-CT. Palliative treatment was undertaken in 21.6% (196 of the patients), and consisted mainly of tumour resection in patients with metastases. The tumour was unresectable in 3% (27 of patients) in whom colostomy or intestinal by-pass was the preferred procedure. Among these, there were 5 patients affected by unresectable colon cancer without distant metastases who underwent diversion colostomy or intestinal by-pass Multivisceral resections were performed in 14.3% of the patients (129/902). The single organ-sites mainly involved in multivisceral resections were the genitourinary system (9.4%), and the small bowel (0.9%).

**Table 1:** Type of surgical procedures performed in 902 patients affected by colorectal cancer

	***COLON N(%)***	***RECTUM N(%)***	***MULTICENTRIC N(%)***	***HNPCC N(%)***
Anterior resection of rectum	-	304	-	-
Right colectomy	201 (42.3)	-	-	-
Left colectomy	167 (35.0)	-	-	-
Segmental resection	79 (16.7)	-	-	6 (75.0)
Abdomino-perineal resection	-	73 (18.0)	-	-
Subtotal colectomy	6 (1.2)	-	11 (91.7)	2 (25.0)
Hartmann resection	8 (1.7)	6 (1.5)	-	-
Diversion colostomy	3 (0.6)	11 (2.7)	-	-
Transanal excision	-	12 (2.9)	-	-
Colonic by-pass	10 (2.1)	-	-	-
Explorative laparotomy	2 (0.4)	-	1 (8.3)	-

**Total**	**476 (100)**	**406 (100)**	**12 (100)**	**8 (100)**

Mean intraoperative blood loss was 300 ± 267 mL (range 100–2200 mL) and rectal cancer surgery was significantly associated with increased intraoperative bleeding (212 ± 187 *vs *376 ± 308 mean blood loss for colonic *vs *rectal resections respectively; p = 0.02). Sixteen patients were intraoperatively transfused (1.8%) with a mean of 1046 ± 867 mL of packed red blood cells (range 500–3000 mL).

### Pathology

The stage distribution of the tumours treated is shown in Table 2. Colon tumours were mainly UICC stages II, III or IV (83.1%), and rectal tumours were of stages I, II or III (86.2%). Tumour stage was not determined for 24 (2.7%) of all tumours. The radicality of the surgical procedure is shown in Table 3. The rate of R0 resections was 77.4% (n = 699) for all patients, 73% (347/476) for colon cancer, 84.7% (344/406) for rectal cancer, and 66.7% (8/12) for multicentric colorectal cancer (p < 0.0001). "Questionably curative" situations, including R1 resections or any other situation that left doubt about the intended curative character of the surgical procedure, occurred in 0.8% (7 of all patients). In 107 out of 129 patients (82.9%) undergoing multivisceral resection, an R0 situation was obtained.

**Table 2:** UICC tumour stage according to site of primary tumour. Mainly, there were more rectal cancers in UICC stage I and colon cancers in UICC stage IV (p < 0.0001).

	***UICC I***	***UICC II***	***UICC III***	***UICC IV***	***Undetermined***	***Total***
**Colon cancer**	80 (16.8)	131 (27.5)	130 (27.3)	132 (27.7)	3 (0.6)	476 (100)
**Rectal cancer**	97 (23.9)	99 (24.4)	136 (33.5)	53 (13.1)	21 (5.2)	406 (100)
HNPCC*	8 (100)	0	0	0	0	8 (100)
**Multicentric**	1 (8.3)	4 (33.3)	4 (33.3)	3 (25)	0	12 (100)

*HNPCC: hereditary non polyposis colorectal cancer

**Table 3:** Surgical radicality according to site of primary tumour. Mainly, there were more rectal cancer patients undergoing R0 resection than colon cancer patients (p < 0.0001).

	***R0 resection***	***R1+R2 resection***	***Non resected***	***Total***
**Colon cancer**	339 (71.2)	121 (25.4)	16 (3.4)	476 (100)
**Rectal cancer**	344 (84.7)	53 (13.1)	9 (2.2)	406 (100)
HNPCC*	8 (100)	0	0	8 (100)
**Multicentric**	8 (66.7)	3 (25)	1 (8.3)	12 (100)

*HNPCC: hereditary non polyposis colorectal cancer

### Morbidity and mortality

Morbidity (Table 4) was documented in 38% of all patients (343/902), 37.8% (180/476) of colon cancer patients, 37.2% (151/406) of rectal cancer patients, 50% (4/8) of HNPCC, and 66.7% (8/12) of multicentric colorectal cancer patients (p = 0.19). The mean intraoperative blood loss for patients who developed or not postoperative complications was 388 ± 384 *vs *254 ± 164 respectively (p = 0.018). The rates of anastomotic leakage were 7% in all 778 patients who had an anastomosis, 5.5% (26/456) for colon patients, 10.4% (33/305) for rectum patients, p = 0.01. Wound infections occurred in rectal cancer patients with a frequency of 20% (81/406) and in those with colon cancer in 19.3% (92/476). Complications were observed equally often in standard and multivisceral resections (37.5% vs. 41.1%, p = 0.44). Nineteen patients required reoperation (13 for anastomotic leakage; 2 for small bowel obstruction; 3 for wound dehiscence, and 1 for mesenteric ischemia). Reoperation was slightly more frequent after colonic (2.7%; 13/476) than rectal resection (1.2%; 5/406) (p = 0.12), and after multivisceral than standard resection (3.1%; 4/129 vs. 1.9%; 15/773) (p = 0.4). The incidence of perioperative blood transfusions was 10.9% (98 patients who received a mean of 857 ± 792 mL of packed red blood cells) with no significant difference between colon and rectal cancer patients (43/476, 9% for colon *vs. *52/406, 12.8% for rectum; p = 0.072). Patients who underwent multivisceral resection were more likely to be transfused (21/129, 16.3% *vs. *77/773, 10%; p = 0.03). The overall complication rate was associated to perioperative blood transfusions (284/804 patients having complications, 35.3%, among non transfused *vs. *59/98, 60.2% among transfused patients; p < 0.0001).

**Table 4:** Complications following surgery according to site of primary tumour. Anastomotic dehiscence were more common after anterior resection of rectum than after surgery for colonic cancer.

	***Colon cancer***	***Rectal cancer***	HNPCC*	***Multicentric***	***p***
**Overall complications**	180/476 (37.8)	151/406 (37.2)	4/8 (50)	8/12 (66.7)	**0.19**
**Postoperative haemoperitoneum**	3/476 (0.4)	2/406 (0.5)	0	0	**0.98**
**Anastomotic dehiscence**	26/456 (5.5)	33/305 (10.4)	2/8 (25)	0	**0.01**
**Abdominal abscess**	17/476 (3.6)	6/406 (1.5)	0	2/12 (16.7)	**0.006**
**Wound complications**	92/476 (19.3)	81/406 (20)	3/8 (37.5)	4/12 (37.6)	**0.39**
**Pneumonia**	16/476 (3.4)	4/406 (1)	0	4/12 (37.3)	**0.025**
**Ileus**	7/476 (1.5)	2/406 (0.5)	0	1/12 (8.3)	**0.051**
**Urinary tract infection**	8/476 (1.7)	15/406 (3.7)	0	1/12 (8.3)	**0.16**
**CVC§infection**	19/476 (4.0)	17/406 (4.2)	0	1/12 (8.3)	**0.82**
**Cardiovascular**	9/476 (1.9)	5/406 (1.2)	0	2/12 (16.7)	**0.001**
**Others minor medical complications**	8/476 (1.7)	8/406 (2.0)	0	0	**0.92**

The 30-day mortality rate was 0.8% (7/902) for all patients. Mortality was 1.3% for colon cancer (6 patients), and 0% for rectal cancer (p = 0.023). There was no difference in the mortality rate between standard and multivisceral resections (0.6%, 5/773 vs. 1.6%, 2/129); p = 0.28). Peroperative mortality rate was significantly correlated with the radicality of the surgical procedure where 0.1% mortality rate (1 patient) was noted for R0 resections, 2.8% (5 patients) for R1+R2 resections and 3.8% (1 patient) for unresected patients (p < 0.0001).

### Tumour recurrence

Patterns of recurrence are represented in table 5. Distant recurrence was the main focus of recurrence both in colon and in rectal cancer. However, local recurrence was significantly more common in rectal cancer patients, associated or not with distant recurrence in 6.1% (29/476) colon; 14.0%, (57/476) rectum (p < 000.1). The total rate of distant metastases into the liver or lungs were resected whenever possible; otherwise they were treated systematically or regionally (liver).

**Table 5:** Patterns of recurrence in 698 patients undergoing R0 resection for colorectal cancer according to site of primary tumour. Local recurrence were significantly more common in rectal cancer patients (p < 0.0001).

	***Local recurrence***	***Local+distant recurrence***	***Distant recurrence***	***Total***
**Colon cancer**	18 (5.2)	11 (3.2)	125 (36.1)	346 (100)
**Rectal cancer**	37 10.8	20 (5.8)	56 (16.3)	344(100)
**Multicentric**	2 (25)	1 (12.5)	4 (50)	8 (100)

Disease-free survival rate for the 699 R0 patients was 73% and 72% at 5 and 8 years respectively and was significantly influenced by the site of the tumour where 5-year disease-free survival rates for colon, rectum and multicentric tumours were 78%, 68% and 24% respectively (p < 0.0001).

### Overall survival rates

**Figure 1:**
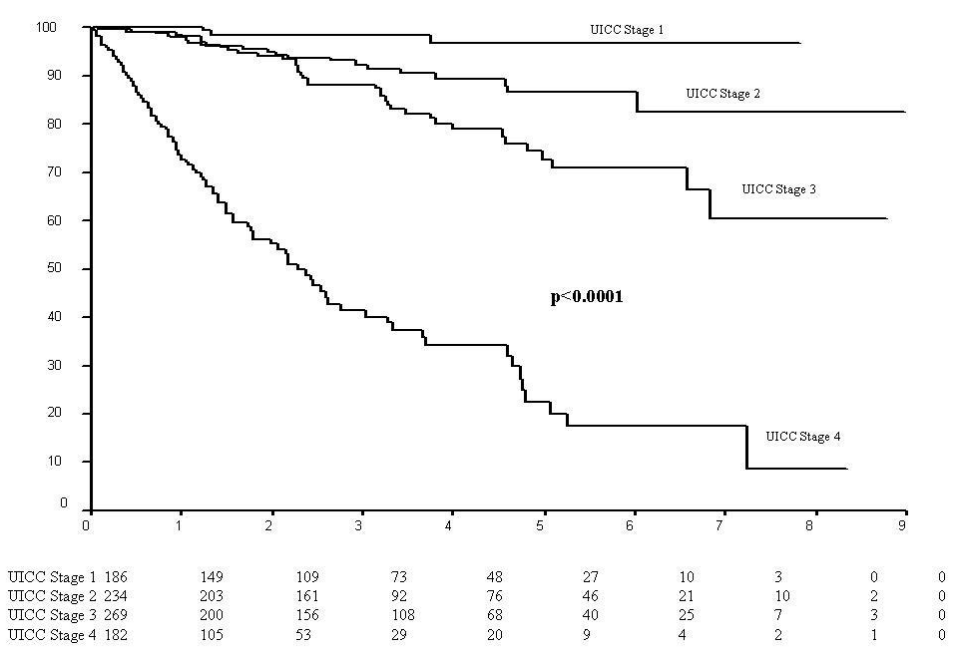
Survival according to UICC tumour stage (7 patients who died peroperatively were excluded from the analysis).

Five- and 8-years overall survival rates were 71% and 61% respectively (total cases) and were significantly dependent on tumour stage (Figure 1).

We decided to perform an univariate analysis on overall survival assessing UICC tumour stage, tumour site (colon vs. rectum vs. multicentric), sex, age (> or <65 years), adjuvant or neoadjuvant treatments, radicality of surgery (R0 vs. R1 vs. R2), postoperative anastomotic leak, perioperative blood transfusions.

For all patients, 5-year survival rates were 97% (UICC I), 87% (UICC II), 73% (UICC III), and 22% (UICC IV), differing significantly (p < 0.0001). The T, N, and M categories significantly influenced survival. Five-year survival rates for T1, T2, T3 and T4 cases were respectively 96%, 87%, 73% and 45% (p < 0.0001). N0 significantly differed from any other N category, where 5-year survival rates for N0, N1 and N2 patients were 86%, 67% and 43% respectively (p < 0.0001) (log-rank test).

Overall 5-year survival rates for patients affected by colon, rectal, or multicentric tumour were 69 vs. 73 vs. 63 respectively (p = 0.061). Neither sex or age >65 year affected overall survival (p = 0.72 and p = 0.12 respectively), nor did the occurrence of an anastomotic leak, (p = 0.85).

**Figure 2:**
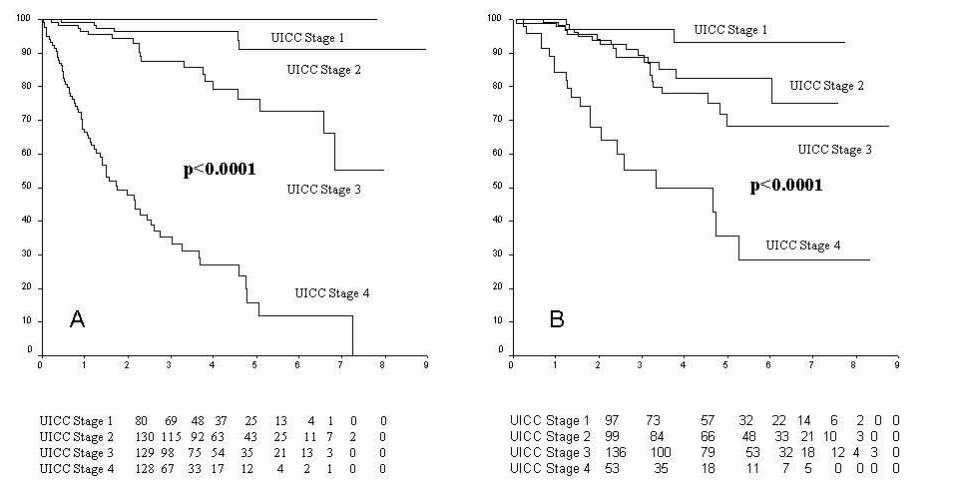
**(a) **Survival according to UICC tumour stage for colon cancer patients undergoing surgery. (b) Survival according to UICC tumour stage for rectal cancer patients undergoing surgery.

For colon cancer (Figure 2a), the 5-year survival rates were 100% (UICC I), 91% (UICC II), 76% (UICC III), and 16% (UICC IV). In the group of UICC T3N0 colon-cancer patients we registered a slight but not significant better survival (92% vs. 80% at 5 years; p = 0.58) among those who underwent adjuvant chemotherapy.

Rectal cancers (Figure 2b) showed 5-year survival rates of 93% (I), 83% (II), 68% (III), 36% (IV), with significant differences (p < 0.0001). Rectal cancer patients undergoing neoadjuvant radiotherapy had a similar overall survival in comparison to patients undergoing postoperative radiotherapy (82% vs. 78% respectively; p = 0.13).

**Figure 3:**
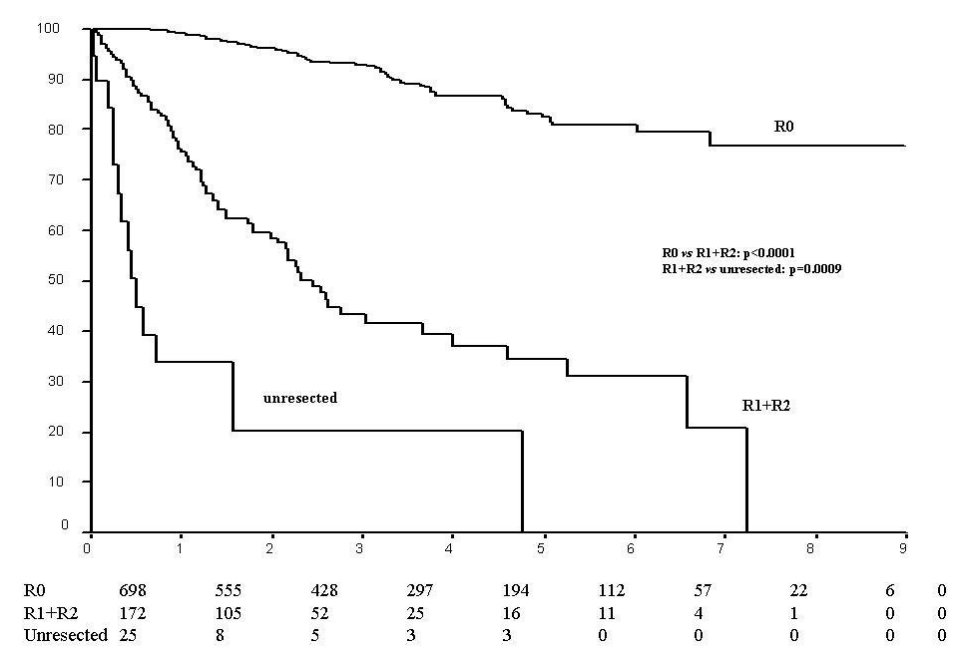
Survival according to radicality of surgery.

The 5-year overall survival showed a marked difference in R0, R1+R2 resection patients and unresected patients with a survival of 82%, 35% and 0% respectively, where a significant difference was noted between R0 vs R1+R2 cases (p < 0.0001) and R1+R2 vs. unresected cases (p = 0.0009) (Figure 3). The influence of any subsequent adjuvant, first line or palliative treatment was not analysed in detail and consisted of heterogeneous groups.

Perioperative blood transfusions didn't influence overall survival (71% and 63% at 5 and 8 years for transfused *vs. *73% and 51% for transfused patients respectively; p = 0.68).

### Multivariate analysis

Factors significant on univariate analysis were matched in a multivariate analysis on overall survival assessing UICC tumour stage, radicality of surgical procedure, and tumour site, where only surgical radicality and UICC tumour stage were associated with outcome (Table 6).

**Table 6:** Multivariate analysis on overall survival for colorectal cancer patients undergoing surgery according to different variables.

	***Hazard ratio***	***95% I. C. §***	**p**
Surgical radicality*	3.000	2.247–4.007	**<0.0001**
**UICC tumour stage**	1.972	1.569–2.477	**<0.0001**
**Tumour site#**	0.851	0.637–1.137	**0.274**

§I. C.: confidence interval

*R0 vs R1+R2 vs unresected patients

#Colon vs rectum vs multicentric cancer

## Discussion

The present study showed that a uniform policy of treatment could provide exceptionally low postoperative mortality rates (0.8%) and excellent results in terms of long-term survival rate (71% at 5 years) in an unselected and consecutive series of colorectal cancer patients who underwent surgery. The ability to remove the primary tumour also in presence of adjacent organ infiltration was one of the main cause of success in our series.

Most colorectal cancers referred to surgical units can be resected, as seen by our overall tumour-resection rate of 97%. This seems remarkable because the prognosis for patients with unresectable tumours was even worse than after palliative resection. However, the institutional resection rate is influenced by both the surgeon and patient factors and can therefore never be taken absolutely or be regarded as a quality-control parameter.

The group of patients with synchronous multicentric colorectal cancer (in our series 1%), is usually not referred to in reports of colorectal-treatment outcome [12] , whereas metachronous colorectal cancer occurs in 0.5% to 4.0% of cases [13,14]. The reported rate of synchronous multicentric colorectal cancer is 2% to 10% and occurs in older patients, mainly in the sigmoid loop and in 30% of cases in nonadjacent colon segments [15]. Synchronous colorectal cancers are usually classified according to site with most advanced-stage tumour and do not show any prognostic differences compared to single-site tumours. However, their existence supports the need for complete staging with total colonoscopy and biopsy of all suspected lesions preoperatively. If total preoperatively colonoscopy cannot be performed, it should be done not later than 3 months postoperatively. Whenever possible, endoscopic polypectomy should be performed prior to surgery.

We registered a morbidity of 38%, and a mortality of 0,8% during the past 9 years of CRC surgery. The apparently high incidence of postoperative overall complication rate may be due to the accurate monitoring and registration of late complications as suggested by others [16]. Conversely, the perioperative mortality figure is quite low in comparison to those reported in literature for elective operations [17]. A possible explanation is that, as recently demonstrated by Billingsley *et al*. [18] , very high surgeon volumes are associated with a reduction in surgical complications. However, the association between increasing hospital volumes and postoperative mortality appears to derive mainly from a full spectrum of clinical services that may facilitate the prompt recognition and treatment of complications. Our findings led us to conclude that although increasing surgeon volumes may decrease complications, decreasing complications is not the major mechanism by which practice volumes decrease postoperative mortality. In fact, minimizing postoperative mortality is associated primarily with systems that provide increased safety in the postoperative period. Our reported 7% rate of anastomotic leakage (5,5% colon, 10,4%rectum) is within the reported range [19]. We prefer stapled anastomoses. Some authors reported a strong relationship between postoperative anastomotic leakage and long-term survival [20]. These findings were not confirmed by our study.

Long-term survival after resection of CRC depends on tumour stage and radicality of the surgical procedure. We observed overall 5-year survival rates of 82% (R0), 35% (R1+R2), 0% (unresectable) in CRC patients. Others reported similar results [19,21-24]. Therefore, the main aim of any surgical procedure is to obtain an R0 resection and to circumvent unresectability even if multivisceral resections are necessary. Multivisceral resections were performed in 14,3% of our patients. This slightly higher rate compared to other reported rates [25-27], is explained by the relatively high number of rectal cancer patients in our Division. Survival rates similar to those for standard resections were observed with multivisceral resections, as in other reports [27-29], so long as R0 resections resulted. However, in contrast to others [30] , we have also seen an indication for multivisceral primary tumour removal when resectable hepatic metastases were found or in patients who were suitable to undergo neoaadjuvant chemotherapy. The prognosis of patients with resectable liver metastases is potentially curative, we observed response rate in 10 patients out of 27 who underwent neoadjuvant chemotherapy for liver metastses from CRC (unpublished data). In these selected patients we observed a 5-year survival rate of 64% following hepatic surgery.

Local tumour recurrence is significantly influenced by surgical technique but occurred in only 2% of cases according to our analysis. We found a significantly higher disease-free survival rate for colon cancer patients than rectal cancer patients: this was probably due to the relatively higher prevalence of local recurrence in the latter group. Despite these findings, overall survival was not different between the two subgroups of patients. Salvage abdominoperineal resection for recurrence following low anterior resection of the rectum was a feasible option with potentially curative results. In fact, if local recurrence is resectable, and is found in combination with resectable single-organ metastses (liver or lung) it should be treated by resection if morbidity is acceptable. Since distant metastases and local recurrence occur in 4,5% of CRC patients and relapse can be prevented at least in part by radiotherapy, chemotherapy, or both, stage dependent adjuvant treatment has been recommended [31-33]. Adjuvant therapy for CRC increased the 5-year survival in randomized studies from 43% to 50% (without adjuvant therapy), to 63% to 70% (with adjuvant therapy) [31,32,34-36]. In our series, adjuvant treatment for T3N0 colon cancer patients failed to show any advantages in term of long-term overall survival. In fact, indications for adjuvant treatment in stage II colorectal cancer are somewhat little established [37]

Indications for adjuvant therapy are currently seen for colon-cancer T independent if the patient is affected by N positive locally advanced or M+ disease.

Overall survival did not differ in our series for rectal-cancer patients who underwent preoperative vs. postoperative radiotherapy. In a recent review authors reported that preoperative radiotherapy improves local recurrence, overall mortality, but does not increase in sphincter sparing procedure rates [38]. However, the benefit of preoperative vs. postoperative radiotherapy remains unclear and debated. Hopefully, randomised controlled trials, which utilise these alternative clinical end points, will in future determine the precise percentages of the effect of different chemoradiation schedules on disease-free and overall survival for rectal cancer patients.

We found a correlation between perioperative blood transfusions and postoperative complication. This was probably due to the fact that patients transfused were at risk for their poor general conditions rather than for an independent effect of blood transfusion itself. We found no relationship between perioperative blood transfusions and long-term survival as reported by others [39].

The usefulness of postoperative surveillance programs has not been clarified yet. It seems likely that early detection of recurrence should improve patients' prognosis [40]. We adopted a uniform policy of follow-up over time with a growing use of PET scan over the last years when recurrence is suspected and the patient could benefit from radical resection of the recurrent disease. This is supported by other studies recently published [41,42].

## Conclusion

In conclusion, our study confirmed that surgery for colorectal cancer is safe (mortality <1%). The main goals remain correct staging of tumour and R0 resection. Multivisceral resections result in morbidity and survival rates comparable to those for standard resections and neoadjuvant treatment in these cases may be necessary.

For patients with R0 resections, adjuvant treatment should be applied on the basis of common recommendations [30] , in special clinical situations (advanced disease, obstruction and/or perforation), and best of all in controlled studies. Despite all the potential and above-mentioned drawbacks of a follow-up and documentation system maintained for more than 10 years, we further conclude from our data that it is a useful tool for surgical quality control.

In addition, it provides a considerably valid institutional basis on which new clinical trials can be planned that will answer open questions about minimal-access-robotic assisted surgery, neoadjuvant treatment, multimodal therapy for colorectal cancer.

## Non-financial competing interests

There are not competing interests (political, personal, religious, ideological, academic, intellectual, commercial or any other) to declare in relation to this manuscript.

## Authors' contributions

**BA**: Assisted in the format and design of the paper

**AC**: Critical review and design of the paper

**EB**: Data analysis

**MB, F O**: Radiologists

**RO**: Radiotherapist

**MGZ, NF**: Oncologists

**MV**: Anaesthesiologist

**AS**: Histopathology evaluation

All authors read and approved the final manuscript
